# RNA sequencing transcriptomics and metabolomics in three poultry breeds

**DOI:** 10.1038/s41597-023-02505-4

**Published:** 2023-09-07

**Authors:** Qidong Zhu, Yuanli Cai, Chuanpi Xiao, Linglian Kong, Xue Pan, Bochen Song, Zhigang Song

**Affiliations:** 1 Key Laboratory of Efficient Utilization of Nongrain Feed Resources, College of Animal Science and Technology, Shandong Agricultural University, Taian, Shandong 271018 China; 2 College of Life Science, Qilu Normal University, Jinan, Shandong 250200 China; 3 Precision Livestock and Nutrition Unit, Gembloux Agro-Bio Tech, University of Liège, Gembloux, 5030 Belgium

**Keywords:** Animal physiology, Transcriptomics, Metabolomics

## Abstract

Chickens are remarkably versatile animals that are used as model organisms for biomedical research. Here, we performed metabolomic and RNA sequencing (RNA-Seq) transcriptomic analyses of the hypothalamus, liver tissue and serum of poultry with different genetic backgrounds, providing detailed information for hypothalamus and liver tissue at the transcriptional level and for liver tissue and serum at the metabolite level. We present two datasets generated from 36 samples from three poultry breeds using high-throughput RNA-Seq and liquid chromatography coupled with mass spectrometry acquisition (LC/MS). The transcriptomic and metabolomic data obtained for poultry of different genetic backgrounds will be a valuable resource for further studies on this model organism.

## Background & Summary

Layer and broiler chickens have undergone extensive selective breeding for specific purposes, resulting in significant differences in various aspects such as feed intake, growth efficiency, body composition, and behavior. It is observed that broilers consume approximately twice the amount of feed compared to layers, and they also spend a higher proportion of their time feeding^1–3^. Moreover, the feed conversion ratio is better in broilers. Numerous studies have shown that body weight is higher in broilers than layers at the same day after hatching^4^. The hybrid chicken named “817” in China was developed as a cross breed between white-feather male broilers (parental generation) and brown-feather female layers (commercial stock). This hybrid chicken has a relatively distinct feeding and growth model. We assumed that the comparation of transcriptomics and metabolomics characters of these three breeds could provide some clues for the clarifying of molecular mechanisms in feed intake and energy regulation.

The energy level in the diet regulates nutritional status and maintains energy homeostasis and appetite in birds^5^.The regulation of appetite is affected by a variety of factors, including regulation by the central nervous system and the peripheral nervous system. AMP-activated protein kinase (AMPK) was activated under conditions of energy depletion, acts as a cellular energy sensor and regulator in the central nervous system and peripheral organs^6^. The peripheral nervous system transports satiety signals and nutrient signals to the central nervous system, and the hypothalamus sends out signals that stimulate or suppress appetite through the perception of nutrition, energy and environmental conditions, thereby achieving complex regulation of feed intake. However, the underlying mechanisms remain incompletely understood among poultry with different genetic backgrounds.

Furthermore, a wealth of scientific research has firmly established a strong correlation between factors such as ghrelin, obestatin, leptin, and the occurrence of obesity as well as type 2 diabetes^7–9^. Notably, features tied to appetite regulation, lipid metabolism, and inflammation have been found to be heritable in individuals with obesity^10^. Additionally, genetic factors may render certain individuals more susceptible to obesity by altering the central and peripheral mechanisms that regulate energy balance and homeostasis^10^. In parallel, the impact of appetite-regulating hormones like ghrelin, obestatin, and leptin on body weight and appetite modulation in chickens has been extensively documented^11,12^. Consequently, these findings may offer valuable insights for the investigation of obesity in humans.

## Methods

This study was approved by Shandong Agricultural University and was conducted in accordance with the Guidelines for Experimental Animals of the Ministry of Science and Technology (Beijing, China). The sampling for our experiment was conducted on December 18, 2020. Subsequently, transcriptomic and metabolomic analyses were initiated on April 15, 2021, with meticulous attention to detail and rigorous protocols. Following the completion of the respective assays, data analysis for the omics datasets was promptly performed.

### Sample collection

Broiler birds (Arbor Acres Plus), layer birds (Hy-Line Brown) and hybrid birds (chicken hybrid 817) were obtained on the day of hatching from the animal experiment station of Shandong Agricultural University (Taian, China). Eggs were provided by a commercial source, and the chicks that hatched from them were sexed, tagged and individually weighed. Only male chicks were employed in this study. The samples were collected and subsequently frozen using liquid nitrogen and stored at −80 °C for further analysis.

### Total RNA isolation

Total RNA was extracted from chicken tissues using TRIzol (Invitrogen 15596018) according to the manufacturer’s instructions. For the quality control assessment of the RNA samples, we employed theilent Bioanalyzer 2100 System (Ag Technologies, CA, USA). This allowed us to ensure the integrity and reliability of the RNA samples before subsequent analysis.

### Transcriptome

High-throughput sequencing was performed by BGISEQ (BGI genomics). For RNA-seq analysis, six biological replicates were performed. The raw sequencing data contained reads with low quality, adapter contamination, and an excessive proportion of N base. It was imperative to remove these problematic reads prior to data analysis to ensure the reliability of the results. Clean reads were mapped to the reference transcriptome using HISAT (hierarchical indexing for spliced alignment of transcripts) and Bowtie2, followed by using RSEM to estimate the gene expression levels in each sample. Differential gene expression analysis based on DESeq 2 was conducted using the methodology described by Michael *et al*.^13^. Genes with an absolute fold change of log2-transformed values ≥1 and a Q-value threshold ≤0.05 were deemed as differentially expressed genes. To evaluate the correlation of gene expression between each pair of samples, the Pearson correlation coefficient was computed for all gene expression values. Only Pearson correlation coefficients >0.80 were utilized for the analysis of within-group comparisons, indicating excellent reproducibility. Thus, thorough data processing and analysis methodologies were employed to ensure the accuracy and validity of the findings.

### Metabolomic

Metabolomic analysis was carried out using established methods^14^. Tissue samples weighing 25 mg were extracted by adding 800 μL of a pre-cooled extraction reagent (methanol: acetonitrile: water, 2:2:1, v/v/v). Internal standards mix 1 and internal standards mix 2 were added to ensure sample preparation quality control. The samples were homogenized for 5 minutes using a Tissue Lyser, followed by sonication for 10 minutes and incubation at −20 °C for 1 hour. After centrifugation at 25000 rpm for 15 minutes at 4 °C, the supernatant was transferred for vacuum freeze drying. The metabolites were then resuspended in 200 μL of 10% methanol, sonicated for 10 minutes at 4 °C, and centrifuged at 25000 rpm for 15 minutes. The resulting supernatants were transferred to autosampler vials for LC-MS analysis. To assess the reproducibility of the LC-MS analysis, a quality control (QC) sample was prepared by pooling the same volume from each sample.

For untargeted metabolomics analysis, LC-MS/MS technology with a high-resolution mass spectrometer Q Exactive (Thermo Fisher Scientific, USA) was used. Data collection was conducted for both positive and negative ions to maximize metabolite coverage. Compound Discoverer 3.1 software (Thermo Fisher Scientific, USA) was employed for LC-MS/MS data processing.

The separation and detection of metabolites were performed using a Waters 2D UPLC system (Waters, USA) coupled to a Q-Exactive mass spectrometer (Thermo Fisher Scientific, USA) equipped with a heated electrospray ionization (HESI) source. The Xcalibur 2.3 software program (Thermo Fisher Scientific, Waltham, MA, USA) controlled the analysis. Chromatographic separation was carried out on a Waters ACQUITY UPLC BEH C18 column (1.7 μm, 2.1 mm × 100 mm, Waters, USA) maintained at 45 °C. In the positive ionization mode, the mobile phase comprised 0.1% formic acid (A) and acetonitrile (B), while in the negative mode, 10 mM ammonium formate (A) and acetonitrile (B) constituted the mobile phase. The gradient conditions were as follows: From 0 to 1 minute, the mobile phase consisted of 2% B, from 1 to 9 minutes, the mobile phase composition varied from 2% to 98% B, at 9 to 12 minutes, the mobile phase contained 98% B, at 12 to 12.1 minutes, the composition changed from 98% B to 2% B, and from 12.1 to 15 minutes, the mobile phase consisted of 2% B. The flow rate during the analysis was maintained at 0.35 mL/min, while injection volume was set at 5 μL.

Moreover, specific mass spectrometric settings were employed for the positive and negative ionization modes. The spray voltage for the positive mode was set at 3.8 kV, whereas for the negative mode, it was set at −3.2 kV. Additionally, the sheath gas flow rate was maintained at 40 arbitrary units (arb), and the auxiliary gas flow rate was set at 10 arb. To ensure optimal performance, the auxiliary gas heater temperature was controlled at 350 °C, and the capillary temperature was maintained at 320 °C.

In order to provide more reliable experimental results during instrument testing, the samples are randomly ordered to reduce system errors. A QC sample is interspersed for every 10 samples. Before establishing the metabolite PCA (principal component analysis) model, log2 conversion was performed on the data, and the Pareto scaling method was used to scale the data. A combination of multivariate statistical analysis and univariate analysis (fold-changes (FC) and T tests (Student’s t test)) was used to screen metabolites showing differences between groups. Differential metabolite screening was performed according to the following conditions: VIP ≥1, fold-change ≥1.2 or ≤0.83, *p* value <0.05 for the first two principal components of the PLS-DA (Partial least squares-discriminant analysis) model.

By employing these precise settings and parameters, we aimed to ensure accurate and comprehensive metabolomic analysis of the samples.

## Data Records

Raw sequencing data have been deposited into the NCBI sequence read archive (SRA) database under accession number PRJNA925883^15^.

The raw LC‒MS data files in.raw formats, were deposited and are publicly available at the MassIVE repository (MSV000091707)^16^. The correlation between the two omics datasets can be found in Supplementary Table 1.

The figshare database encompasses two main sections, namely transcriptomic and metabolomic data^17^. Within the transcriptomic section, there are comprehensive grouping information and quality control analysis tables available for transcriptomes of the liver and hypothalamus. Moreover, this section includes additional supplementary files, such as PCA analysis, sample correlation analysis, expression distribution through Box plots, as well as highly detailed information pertaining to Reads alignment and Reads filter processes. Correspondingly, the metabolomic section presents data on experimental grouping information, quality control measures, and comprehensive statistical analyses encompassing PCA and PLSDA (Partial Least Squares Discriminant Analysis). Additionally, this section offers partial difference analysis results files for liver and serum metabolomes. These extensive datasets provide thorough insights into the transcriptional and metabolic profiles, enabling a comprehensive evaluation of the experimental findings.

## Technical Validation

High-throughput RNA-Seq generated a total of 42.84 gigabytes (GB) of clean data from 36 samples collected from two tissues (hypothalamus and liver) of poultry with three different genetic backgrounds (broiler, layer, and hybrid chickens).

The amount of clean data obtained from each sample reached 1.19 Gb, and the Q30 base percentage was 92.39% or higher (Table 1). A total of 17052 and 16409 genes were identified in the hypothalamus and liver, respectively. We used HISAT to align the clean reads to the designated chicken reference genome. The percentages of successfully mapped reads ranged between 90.83 and 93.35% (Table 1). Differentially expressed genes were selected according to the comparison results and their expression levels in different samples.

**Table 1:** RNA-seq read statistics.

Sample	Clean Reads	N Read Num	Q20%	Q30%	HISAT Mapped %	Bowtie2 Mapped %	HISAT Uniq Mapped %	Bowtie2 Uniq Mapped %
Hypothalamus								
Broiler1	23795369	105506	98.29	94.85	92.78	64.93	91.83	62
Broiler2	23787825	113973	98.19	94.63	92.66	60.37	91.72	57.64
Broiler3	23629939	106009	98.28	94.85	92.13	60.73	91.11	57.81
Hybrid1	23755420	118209	98.13	94.49	92.24	59.19	91.19	56.28
Hybrid2	23769273	114293	98.03	94.23	91.96	55.84	90.98	53
Hybrid3	23756425	108241	98.21	94.66	92.44	62.16	91.46	59.23
Hybrid4	23746986	117332	97.98	94.09	91.78	64.33	90.74	61.44
Hybrid5	23778189	116095	98.02	94.15	92.47	64.55	91.5	61.74
Hybrid6	23774207	114751	97.92	93.87	91.91	64.49	90.88	61.45
Layer 1	23786405	99412	98.37	95.1	92.86	62.7	91.87	59.75
Layer 2	23784108	114674	98.03	94.18	92.13	63.46	91.21	60.67
Layer 3	23769245	116269	98	94.13	92.18	63.43	91.18	60.62
Layer 4	23785986	111860	98.08	94.33	92.32	63.75	91.38	61
Layer 5	23776064	114888	98.02	94.14	92.52	63.09	91.6	60.42
Layer 6	22879026	88025	98.59	95.68	93.35	64.23	92.43	61.46
Liver								
Broiler1	23752679	46014	97.32	92.71	91.53	60.69	90.35	58.25
Broiler2	23754844	48857	97.35	92.78	91.56	59.28	90.44	56.82
Broiler3	23820037	47388	97.47	93.05	92.05	66.3	90.94	63.97
Broiler4	23770474	48961	97.31	92.71	91.74	56.21	90.66	53.94
Broiler5	23807332	48292	97.33	92.73	91.52	61.33	90.44	58.96
Broiler6	23799266	47929	97.2	92.39	91.25	64.7	90.05	62.27
Hybrid1	23795545	46263	97.4	92.91	91.22	58.65	89.9	56.29
Hybrid2	23791190	47703	97.32	92.7	90.83	56.41	89.6	54.2
Hybrid3	23808883	46560	97.52	93.16	91.95	67.84	90.74	65.56
Hybrid4	23812764	44323	97.48	93.03	92.07	63.87	90.82	61.45
Hybrid5	23810895	44730	97.61	93.41	91.94	62.32	90.71	60.01
Hybrid6	23800781	48370	97.44	93	91.64	51.9	90.41	49.67
Layer 1	23759576	43234	97.58	93.29	91.54	58.94	90.22	56.49
Layer 2	23805208	46435	97.3	92.66	90.92	60.55	89.59	58.1
Layer 3	23786110	42103	97.71	93.56	92.4	61	91.19	58.5
Layer 4	23763257	48952	97.3	92.67	91.16	58.02	90.02	55.49
Layer 5	23707306	71960	97.32	92.67	91.06	57.64	89.92	55.66
Layer 6	23747845	71199	97.68	93.59	92.08	63.42	90.86	60.94

**Fig. 1:**
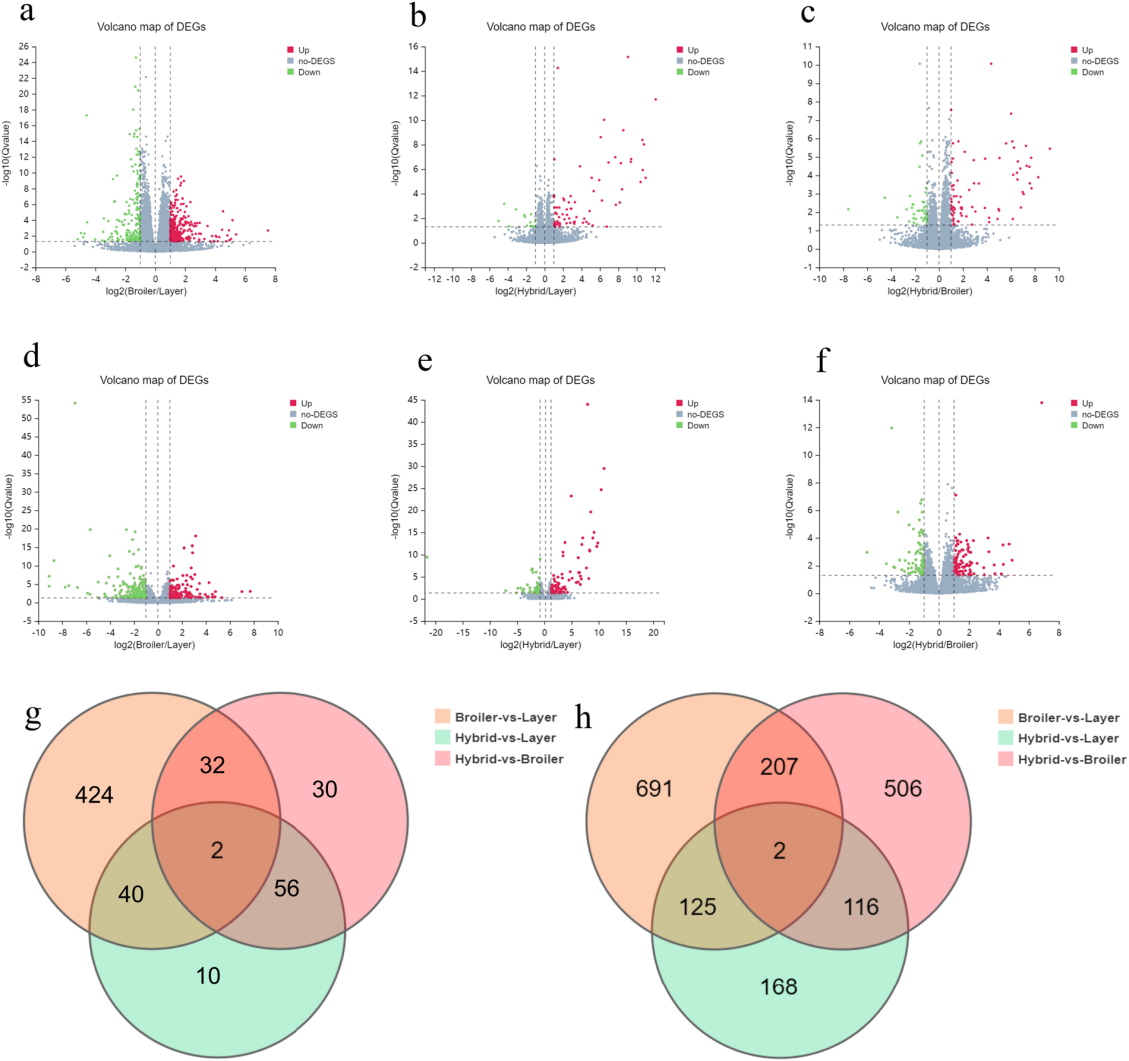
Analysis of significant DEGs between broiler, layer, and hybrid chickens. Volcano diagram of DEGs between broiler, layer, and hybrid chickens in three comparison groups (layer vs. broiler, layer vs. hybrid, broiler vs. hybrid) for the hypothalamus (**a**–**c**) and the liver (**d**–**f**). The X-axis represents the fold change in the difference after log2 conversion, and the Y-axis represents the significance value after log10 conversion. Red represents upregulated DEGs, green represents downregulated DEGs, and gray represents non-DEGs; Venn diagram showing common and unique genes among the three groups in two tissues: hypothalamus (**g**) and liver (**h**).

A total of 560 and 589 differentially expressed genes (DEGs) were identified in the hypothalamus and liver, respectively (Table 2), and the patterns in the hypothalamus patterns are illustrated by the degree of dispersion in the volcano map of DEGs (Fig. 1a–h). A comparison of DEGs (Q value ≤ 0.05 and |log2 (fold change) | ≥ 1) among different groups, as illustrated in a Venn diagram, revealed both common and specific changes in gene expression triggered in the two tissues in the three groups (Fig. 1g,h).

**Table 2:** Pairwise comparison of up and downregulated DEGs between broiler, layer, and hybrid chickens in hypothalamus and liver.

Compare Group	Down	Up	Total
Hypothalamus			
Layer -vs.- Broiler	182	316	498
Layer -vs.- Hybrid	14	70	84
Broiler -vs.- Hybrid	42	78	120
Liver			
Layer -vs.- Broiler	241	187	428
Layer -vs.- Hybrid	49	90	139
Broiler -vs.- Hybrid	82	94	176

**Fig. 2:**
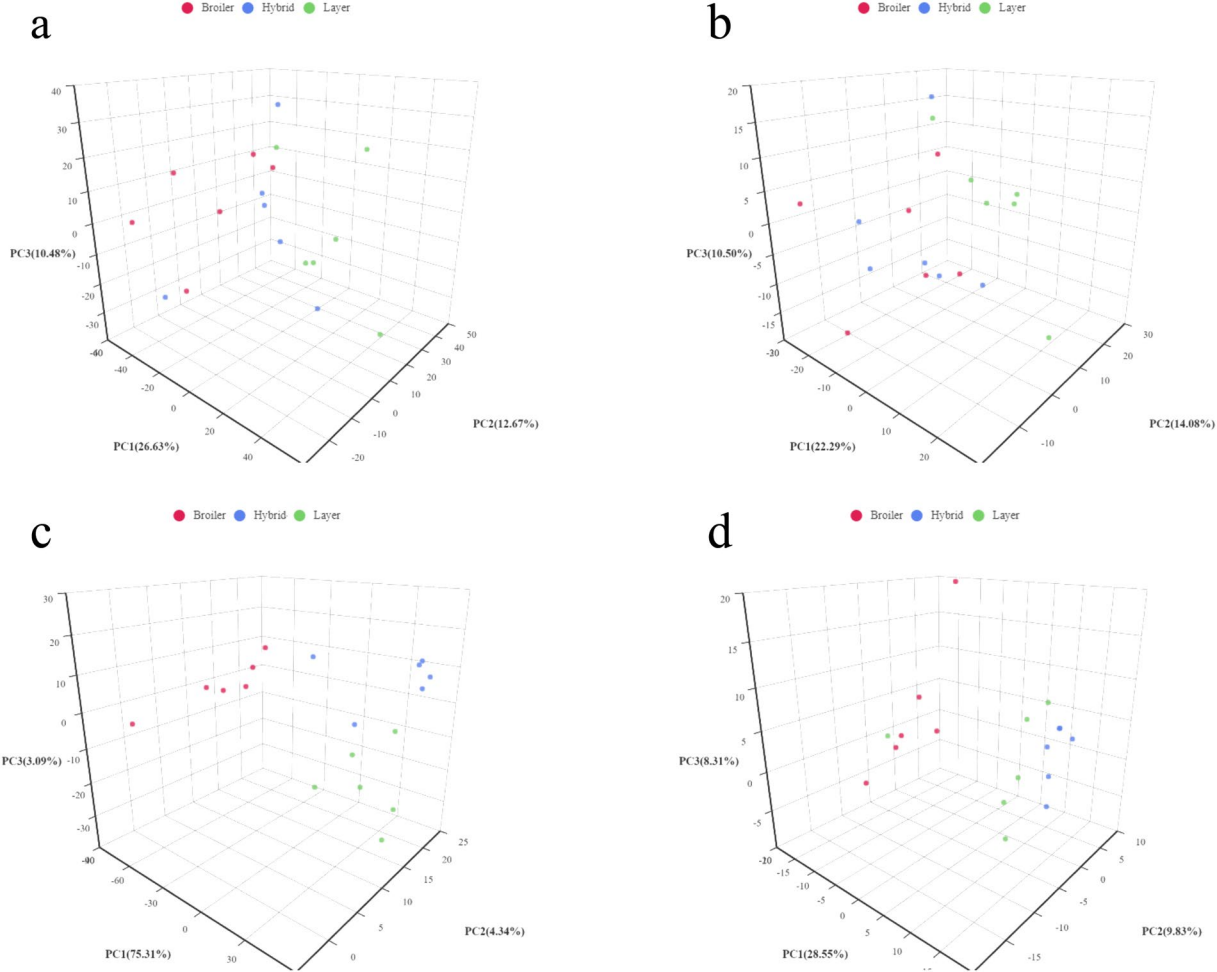
PCA score plot based on all metabolites. The X axis represents the first principal component (PC1), the Y axis represents the second principal component (PC2), and the Z axis represents the third principal component (PC3). Each dot represents a sample, and different groups are labeled with different colors. The number is the score of the principal component, which represents the percentage of the overall variance explained by the specific principal component. (**a**) PCA graphs of the liver metabolome in positive ion metabolomics analysis, (**b**) the liver metabolome in negative ion analysis, (**c**) the serum metabolome in positive ion analysis, (**d**) the serum metabolome in negative ion analysis.

A total of 1,136 pos and 448 neg compounds in liver and 719 pos compounds and 275 neg compounds in serum were identified. We constructed principal component analysis (PCA) models to investigate the correlations between metabolite levels and genetic backgrounds. The PCA model (Fig. 2a–d) represents the distribution and separation trends observed in the broiler, hybrid and layer comparison groups. As shown in Fig. 2c,d, three principal components exhibited obvious clusters within the three groups, whereas between-group comparisons revealed no clusters, indicating that the genetic background was responsible for a majority of the differences between the metabolic profiles in serum.

This study used metabolomics and transcriptomics to reveal related functional genes or genetic markers that affect appetite in poultry, further clarifying the neuroendocrine mechanism of energy metabolism regulation.

### Supplementary information

Supplementary Information

## Data Availability

No custom code was used to generate or process the data described in this manuscript.

## References

[1] Buzala M, Janicki B (2016). Review: Effects of different growth rates in broiler breeder and layer hens on some productive traits. Poultry Science..

[2] Saneyasu T (2011). Neuropeptide Y effect on food intake in broiler and layer chicks. Comparative Biochemistry and Physiology Part A: Molecular & Integrative Physiology..

[3] Wang Y, Buyse J, Song Z, Decuypere E, Everaert N (2016). AMPK is involved in the differential neonatal performance of chicks hatching at different time. General and Comparative Endocrinology..

[4] Druyan S (2010). The effects of genetic line (broilers vs. layers) on embryo development. Poultry Science..

[5] Hu X (2019). Effects of dietary energy level on appetite and central adenosine monophosphate-activated protein kinase (AMPK) in broilers. J Anim Sci..

[6] Huynh MKQ, Kinyua AW, Yang DJ, Kim KW (2016). Hypothalamic AMPK as a Regulator of Energy Homeostasis. Neural Plasticity..

[7] Ahima RS, Saper CB, Flier JS, Elmquist JK (2000). Leptin Regulation of Neuroendocrine Systems. Frontiers in Neuroendocrinology..

[8] Parker JA, Bloom SR (2012). Hypothalamic neuropeptides and the regulation of appetite. Neuropharmacology..

[9] Montégut L, Lopez-Otin C, Magnan C, Kroemer G (2021). Old Paradoxes and New Opportunities for Appetite Control in. Obesity. Trends in Endocrinology & Metabolism..

[10] Xiao Y, Liu D, Cline MA, Gilbert ER (2020). Chronic stress, epigenetics, and adipose tissue metabolism in the obese state. Nutr Metab (Lond)..

[11] Kuo AY, Cline MA, Werner E, Siegel PB, Denbow DM (2005). Leptin effects on food and water intake in lines of chickens selected for high or low body weight. Physiology & Behavior..

[12] Xu P, Siegel PB, Denbow DM (2011). Genetic selection for body weight in chickens has altered responses of the brain’s AMPK system to food intake regulation effect of ghrelin, but not obestatin. Behavioural Brain Research..

[13] Love MI, Huber W, Anders S (2014). Moderated estimation of fold change and dispersion for RNA-seq data with DESeq 2. Genome Biol..

[14] Dunn WB (2011). Procedures for large-scale metabolic profiling of serum and plasma using gas chromatography and liquid chromatography coupled to mass spectrometry. Nat Protoc..

[15] (2023). NCBI Sequence Read Archive.

[16] Zhu Q (2023). MassIVE.

[17] Zhu Q (2023). Figshare.

