# Supplementary material for: RNA sequencing transcriptomics and metabolomics in three poultry breeds
Source: Sci Data. 2023 Sep 7;10:594. doi: 10.1038/s41597-023-02505-4 (PMC10484955; doi:10.1038/s41597-023-02505-4)
Supplement: Supplementary file 1 — Supplementary Information [file 41597_2023_2505_MOESM1_ESM.pdf]

Supplementary Information

RNA sequencing transcriptomics and metabolomics in three poultry breeds

Qidong Zhu[1], Yuanli Cai[2], Chuanpi Xiao[1,3], Linglian Kong[1], Xue Pan[1], Bochen Song[1], Zhigang Song[1]

Affiliations

1. Key Laboratory of Efficient Utilization of Nongrain Feed Resources, College of Animal Science and Technology, Shandong Agricultural University, Taian, Shandong 271018, China
2. College of Life Science, Qilu Normal University, Jinan, Shandong 250200, China
3. Precision Livestock and Nutrition Unit, Gembloux Agro-Bio Tech, University of Liège, Gembloux 5030, Belgium

corresponding author: Zhigang Song (zhigangs@sdau.edu.cn)

Content

Supplementary Table 1

Supplementary Table 1. General information of database depositories

Breed	Sample	Tissue	SRA Accession	MassIVE File Name
Arbor Acres Plus	MLA1	Liver	SAMN32816302	21P00960001_BEHC18_POS/NEG
Arbor Acres Plus	MLA2	Liver	SAMN32816303	21P00960002_BEHC18_POS/NEG
Arbor Acres Plus	MLA3	Liver	SAMN32816304	21P00960003_BEHC18_POS/NEG
Arbor Acres Plus	MLA4	Liver	SAMN32816305	21P00960004_BEHC18_POS/NEG
Arbor Acres Plus	MLA5	Liver	SAMN32816306	21P00960005_BEHC18_POS/NEG
Arbor Acres Plus	MLA6	Liver	SAMN32816307	21P00960006_BEHC18_POS/NEG
Chicken hybrid 817	MLB1	Liver	SAMN32816308	21P00960007_BEHC18_POS/NEG
Chicken hybrid 817	MLB2	Liver	SAMN32816309	21P00960008_BEHC18_POS/NEG
Chicken hybrid 817	MLB3	Liver	SAMN32816310	21P00960009_BEHC18_POS/NEG
Chicken hybrid 817	MLB4	Liver	SAMN32816311	21P00960010_BEHC18_POS/NEG
Chicken hybrid 817	MLB5	Liver	SAMN32816312	21P00960011_BEHC18_POS/NEG
Chicken hybrid 817	MLB6	Liver	SAMN32816313	21P00960012_BEHC18_POS/NEG
Hy-Line Brown	MLC1	Liver	SAMN32816314	21P00960013_BEHC18_POS/NEG
Hy-Line Brown	MLC2	Liver	SAMN32816315	21P00960014_BEHC18_POS/NEG
Hy-Line Brown	MLC3	Liver	SAMN32816316	21P00960015_BEHC18_POS/NEG
Hy-Line Brown	MLC4	Liver	SAMN32816317	21P00960016_BEHC18_POS/NEG
Hy-Line Brown	MLC5	Liver	SAMN32816318	21P00960017_BEHC18_POS/NEG
Hy-Line Brown	MLC6	Liver	SAMN32816319	21P00960018_BEHC18_POS/NEG
Arbor Acres Plus	MHA1	hypothalamus	SAMN32816284	
Arbor Acres Plus	MHA2	hypothalamus	SAMN32816285	
Arbor Acres Plus	MHA3	hypothalamus	SAMN32816286	
Arbor Acres Plus	MHA4	hypothalamus	SAMN32816287	
Arbor Acres Plus	MHA5	hypothalamus	SAMN32816288	
Arbor Acres Plus	MHA6	hypothalamus	SAMN32816289	
Chicken hybrid 817	MHB1	hypothalamus	SAMN32816290	
Chicken hybrid 817	MHB2	hypothalamus	SAMN32816291	
Chicken hybrid 817	MHB3	hypothalamus	SAMN32816292	
Chicken hybrid 817	MHB4	hypothalamus	SAMN32816293	
Chicken hybrid 817	MHB5	hypothalamus	SAMN32816294	
Chicken hybrid 817	MHB6	hypothalamus	SAMN32816295	
Hy-Line Brown	MHC1	hypothalamus	SAMN32816296	
Hy-Line Brown	MHC2	hypothalamus	SAMN32816297	
Hy-Line Brown	MHC3	hypothalamus	SAMN32816298	
Hy-Line Brown	MHC4	hypothalamus	SAMN32816299	
Hy-Line Brown	MHC5	hypothalamus	SAMN32816300	
Hy-Line Brown	MHC6	hypothalamus	SAMN32816301	
Arbor Acres Plus	A1	Serum		21P00950001_BEHC18_POS/NEG

Arbor Acres Plus	A2	Serum	21P00950002_BEHC18_POS/NEG
Arbor Acres Plus	A3	Serum	21P00950003_BEHC18_POS/NEG
Arbor Acres Plus	A4	Serum	21P00950004_BEHC18_POS/NEG
Arbor Acres Plus	A5	Serum	21P00950005_BEHC18_POS/NEG
Arbor Acres Plus	A6	Serum	21P00950006_BEHC18_POS/NEG
Chicken hybrid 817	B1	Serum	21P00950007_BEHC18_POS/NEG
Chicken hybrid 817	B2	Serum	21P00950008_BEHC18_POS/NEG
Chicken hybrid 817	B3	Serum	21P00950009_BEHC18_POS/NEG
Chicken hybrid 817	B4	Serum	21P00950010_BEHC18_POS/NEG
Chicken hybrid 817	B5	Serum	21P00950011_BEHC18_POS/NEG
Chicken hybrid 817	B6	Serum	21P00950012_BEHC18_POS/NEG
Hy-Line Brown	C1	Serum	21P00950013_BEHC18_POS/NEG
Hy-Line Brown	C2	Serum	21P00950014_BEHC18_POS/NEG
Hy-Line Brown	C3	Serum	21P00950015_BEHC18_POS/NEG
Hy-Line Brown	C4	Serum	21P00950016_BEHC18_POS/NEG
Hy-Line Brown	C5	Serum	21P00950017_BEHC18_POS/NEG
Hy-Line Brown	C6	Serum	21P00950018_BEHC18_POS/NEG
